# Crystal Structure of a Coiled-Coil Domain from Human ROCK I

**DOI:** 10.1371/journal.pone.0018080

**Published:** 2011-03-21

**Authors:** Daqi Tu, Yiqun Li, Hyun Kyu Song, Angela V. Toms, Christopher J. Gould, Scott B. Ficarro, Jarrod A. Marto, Bruce L. Goode, Michael J. Eck

**Affiliations:** 1 Department of Biological Chemistry and Molecular Pharmacology, Harvard Medical School, Boston, Massachusetts, United States of America; 2 Department of Cancer Biology, Dana-Farber Cancer Institute, Boston, Massachusetts, United States of America; 3 School of Life Sciences and Biotechnology, Korea University, Seoul, Korea; 4 Department of Biology and Rosenstiel Basic Medical Science Research Center, Brandeis University, Waltham, Massachusetts, United States of America; University of Oulu, Germany

## Abstract

The small GTPase Rho and one of its targets, Rho-associated kinase (ROCK), participate in a variety of actin-based cellular processes including smooth muscle contraction, cell migration, and stress fiber formation. The ROCK protein consists of an N-terminal kinase domain, a central coiled-coil domain containing a Rho binding site, and a C-terminal pleckstrin homology domain. Here we present the crystal structure of a large section of the central coiled-coil domain of human ROCK I (amino acids 535–700). The structure forms a parallel α-helical coiled-coil dimer that is structurally similar to tropomyosin, an actin filament binding protein. There is an unusual discontinuity in the coiled-coil; three charged residues (E613, R617 and D620) are positioned at what is normally the hydrophobic core of coiled-coil packing. We speculate that this conserved irregularity could function as a hinge that allows ROCK to adopt its autoinhibited conformation.

## Introduction

Rho is a small GTPase that upon conversion from its inactive form (GDP • Rho) to active form (GTP • Rho) binds to specific effectors to exert its biological functions. Among the downstream targets of activated Rho are two isoforms of Rho-associated kinase (ROCK), ROCK I/ROKβ/p160ROCK [1], [2], [3] and ROCK II/ROKα/Rho kinase [4], [5], which share 65% sequence identity and 95% sequence similarity at the amino acid level (reviewed in [6]). ROCK is able to regulate the phosphorylation of myosin light chain (MLC) by direct phosphorylation [7] and by inactivation of myosin phosphatase through the phosphorylation of its myosin-binding subunit (MBS) [8]. Although phosphorylation of MLC in smooth muscle is regulated primarily by MLC kinase in a Ca^2+^-dependent manner, ROCK has an important role in agonist-induced Ca^2+^-sensitization in muscle contraction [9]. In non-muscle cells, the Rho/ROCK pathway is involved in stress fiber and focal adhesion formation [2], [10], [11], neurite retraction [12], and cell migration [13]. Increased MLC phosphorylation in non-muscle cells enhances the actin binding and actin-induced ATPase activity of myosin II. The enhanced binding of myosin to actin promotes the bundling of F-actin into stress fibers and the subsequent formation of focal adhesions [14]. ROCK also phosphorylates and activates LIM-kinase, which in turn phosphorylates and inactivates the actin-depolymerizing and severing factor cofilin [15]. As a result, existing actin filaments are stabilized.

Other downstream effectors of Rho signaling include mDia (mouse homolog of Drosophila diaphanous). mDia is a formin molecule that catalyzes actin nucleation and polymerization to produce long, straight actin filaments [16]. ROCK and mDia work together to induce stress fibers [17] and also in regulating formation of the actin contractile ring in mitotic cells [18], [19].

Through its effects on vascular smooth muscle contractility, ROCK has been linked to hypertension [20], vasospasm [21] and bronchial asthma [22]. ROCK is also implicated in non-muscle cancer cell invasion and metastasis [23], [24]. Because the Rho/ROCK regulatory pathway is involved in a variety of diseases, it has become a promising target for therapeutic interventions.

**Figure 1:**
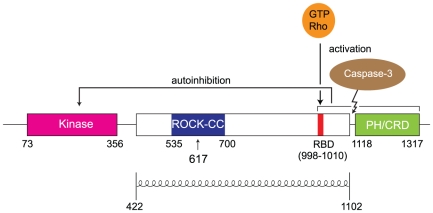
Schematic view of the ROCK I domain structure. In the inactive form, the Rho-binding domain (RBD) and the pleckstrin homology (PH) domain bind to the amino-terminal kinase domain. This autoinhibitory interaction is released either by GTP-Rho binding to RBD, or by caspase-3 cleavage of the PH domain. PH domain contains an internal cysteine-rich domain (CRD). The entire central domain (422–1102) is predicted to be coiled-coil, as indicated. ROCK-CC (in blue) is the solved crystal structure region in this report. The position of residue 617 is indicated with an arrow.

Human ROCK I consists of an N-terminal serine/threonine kinase domain (residues 73–356), a central predicted coiled-coil (residues 422–1102), and a C-terminal pleckstrin homology (PH) domain (residues 1118–1317). The PH domain contains an internal cysteine-rich domain (CRD) that resembles the C1 domain of protein kinase C (Fig. 1). The Rho binding domain (RBD) of ROCK I is located near the end of the central coiled-coil [25]. The C-terminal region of ROCK II (RBD plus the PH domain) has been shown to directly interact with the kinase domain to inhibit its activity [26]. Interaction between GTP-Rho and the RBD disrupts ROCK I's autoinhibition and thus activates the kinase [3]. Cleavage of the ROCK I C-terminal domain by caspase-3 during apoptosis also results in an activated kinase [27], [28].

Crystal structures have been reported for the RBD in complex with activated RhoA [25] and for a large fragment encompassing the kinase domain and a dimerization domain N-terminal to it [29]. In order to obtain a more complete understanding of the structure of this important molecule, we have determined the crystal structure of a stable tryptic fragment of the large central coiled-coil domain of ROCK I (Fig. 1). The structure covers residues 535 to 700 and is a long asymmetric parallel coiled-coil. This region of ROCK I is structurally similar to tropomyosin. The structure also reveals an irregularity in the coiled-coil conformation in which three charged residues (E613, R617 and D620) are positioned in the typically hydrophobic *a* and *d* positions at the core of coiled-coil packing. This E613/R617/D620 bulge appears to locally destabilize the coiled-coil and bend its axis by about 11°. The significance of this irregularity, if any, is unclear. However, we speculate that it might serve as a hinge that enables the C-terminal inhibitory region to bind the N-terminal kinase domain in an autoinhibited conformation.

## Results

### Structure Determination

We obtained crystals of the ROCK coiled-coil domain (ROCK-CC, residues 535–700) in the tetragonal space group *P*4_3_2_1_2. The crystals diffracted anisotropically; we observed strong reflections to beyond 2.3 Å along c* but only to 2.9 Å in the perpendicular plane. The diffraction data were therefore filtered so as to retain only reflections lying within an ellipsoid defined by these resolution limits (see methods and Table 1, 2 and 3). The structure of ROCK-CC was solved by single-wavelength anomalous dispersion (SAD) phasing at the peak wavelength using selenomethionine (SeMet) substituted protein. Density modification resulted in a readily interpretable electron density map in which 52% of side chains were automatically assigned. Iterative model building and refinement produced a model with good statistics and geometry (Table 1). There are four molecules (2 dimers) per asymmetric unit. The final model includes 623 residues and was refined to an *R*_free_ = 28.6% and R = 23.9%. The quality of the electron density map was sufficiently clear for unambiguous fitting of residues 542–693 of chain A, 541–692 of chain B, 535–692 of chain C and 535–693 of chain D. However the quality of the map was poorer for residues 542–552 of chain A, 541–549 of chain B, 541–549 and 566–578 of chain C, and 562–573 of chain D, which have high thermal factors.

**Table 1:** X-ray data collection and refinement.

	ROCK (535–700)	ROCK (535–709)
*Data collection*		
Wavelength, Å	0.97914	0.97950
Space group	P4_3_2_1_2	P1
a, b, c, Å (α, β, γ, deg.)	84.6, 84.6, 340.1	35.1, 63.5, 90.5 (76.6, 89.1, 89.4)
Resolution (a*, b*, c*, Å)	2.92, 2.92, 2.33	2.40, 2.40, 2.40
Unique reflections	69169	27910
Redundancy	30.4 (25.6)	1.9 (1.8)
Completeness, %	67.6 (14.5)	94.3 (71.8)
I/σ	30.7 (13.2)	12.9 (3.2)
R_sym_, %†	12.0 (33.4)	5.5 (21.0)
*Phasing*		
Figure of merit (acentric/centric)	0.46/0.09	
*Refinement*		
Resolution range, Å	42.1−2.33	
R-factor/R_free_ ‡	0.239/0.286	
Bond length deviation, Å	0.009	
Bond angle deviation, °	1.1	
Average B factor, Å^2^	80.0	
Ramachandran plot, %		
Preferred region (outlier)	98.8 (0)	

Values in parentheses are for the outer shell (2.41−2.33 Å) for ROCK(535–700), and (2.49−2.40 Å) for ROCK (535–709).

† *R*_sym_  =  ∑_h_∑_i_| I_i_(h) - <I(h)>| /∑_h_∑_i_I_i_(h), where I_i_(h) is the i^th^ measurement and <I(h)> is the mean of all measurements of I(h) for Miller indices h.

‡ R  =  ∑(| F_obs_| - | F_calc_|)/∑| F_obs_|. *R*_free_ is obtained for a test set of reflections (5.3% of total).

**Table 2:** Anisotropic Data Statistics.

Lower, Å	Upper, Å	Completeness, %	*R*_sym_
50.0	5.02	99.5	6.8
5.02	3.98	100.0	9.4
3.98	3.48	100.0	12.0
3.48	3.16	100.0	21.2
3.16	2.94	100.0	29.6
2.94	2.76	66.8	28.0
2.76	2.62	42.9	32.6
2.62	2.51	30.5	27.0
2.51	2.41	21.7	31.8
2.41	2.33	14.5	33.4

**Table 3:** Data Collection Statistics without Ellipsoidal Filtering.

Lower, Å	Upper, Å	Completeness, %	*R*_sym_
50.0	5.02	99.5	6.9
5.02	3.98	100.0	9.4
3.98	3.48	100.0	12.0
3.48	3.16	100.0	21.2
3.16	2.94	100.0	29.6
2.94	2.76	100.0	41.4
2.76	2.62	100.0	77.1
2.62	2.51	100.0	91.8
2.51	2.41	99.9	0.00
2.41	2.33	98.5	0.00

### Overall Structure

**Figure 2:**
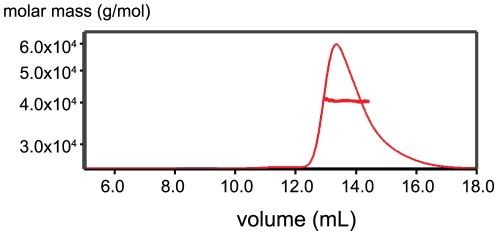
ROCK-CC forms a dimer in solution. Purified ROCK (535–704) was analyzed on a Superdex 200 gel filtration column coupled to a multi-angle light scattering detector. The protein elution profile measured by refractive index is shown as a thin red trace; the horizontal thick red line is measured molar mass (∼40.5 KDa). Theoretical molar mass of a dimer is 40.4 KDa.

**Figure 3:**
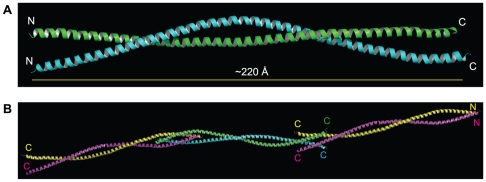
Overall structure of ROCK-CC. (A) Ribbon diagram of the ROCK-CC dimer is shown with chain A in green and chain B in cyan. The parallel coiled-coil homo-dimer is asymmetric. (B) Crystal packing of dimer A/B with dimer C/D. The orientation is that of Fig. 3A turned 90° towards the viewer along the superhelical axis. Chain C is colored in yellow, chain D is in magenta.

ROCK-CC is a dimer in solution as determined by size-exclusion chromatography coupled to multi-angle light scattering (SEC-MALS) [Fig. 2]. The two dimers in the asymmetric unit of ROCK-CC crystal are fundamentally the same, but the dimer of chains A and B is better ordered than that of chains C and D. In the following analysis we concentrate on dimer A/B if not stated otherwise. The structure reveals two long α helices that intertwine to form a two-stranded parallel coiled-coil that is about 220 Å long (Fig. 3A). The total buried accessible surface area is 5054 Å^2^. The helices at either end of the ROCK-CC are splayed apart due to crystal packing (Fig. 3B). The N terminal end of dimer A/B packs with the N terminal end of dimer C/D in a head-to-head manner. Similarly, the C terminal end of dimer A/B packs with the C terminal end of a symmetry mate of dimer C/D in a tail-to-tail arrangement. In the crystal lattice, this packing leads to an infinitely long cable. Obviously, these interactions are not expected to occur in the context of the full-length protein.

While only residues 589–666 are in a parallel coiled-coil conformation in the present structure, we expect that this will be part of a much larger coiled-coil in the intact ROCK I protein. Coiled-coil prediction program COILS [30] assigned residues 422 to 1102 to be coiled-coil (Fig. 1). Crystal packing is likely responsible for terminating coiled-coil conformation in the present structure.

### ROCK-CC Dimer Interface

A canonical left-handed coiled-coil has a periodicity of seven residues over two helical turns, which is called a heptad repeat. These seven residues are commonly designated as (*a*-*b*-*c*-*d*-*e*-*f*-*g*). The interaction between the helices is mediated by the hydrophobic residues at positions *a* and *d* and oppositely charged residues at the positions *e* and *g* [31].

**Figure 4:**
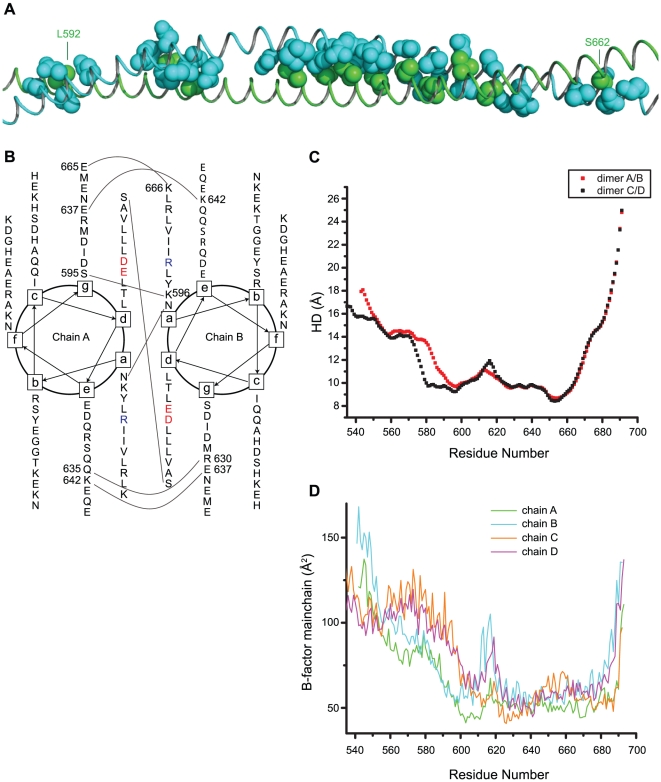
Inter-helical interactions in ROCK-CC. (A) In sphere representation, knobs from chain A (green) pack into holes from chain B (cyan). For clarity, only side chains are shown and knobs from chain B packing into holes from chain A are not displayed. (B) Helical wheel diagram of residues Asn-589 to Glu-665. Interhelical polar interactions are indicated by a line connecting paired residues. E613 and D620 at the *d* positions are colored red. R617 at the *a* position is colored blue. (C) Inter-Helical distance (HD) for two structures. Due to crystal packing, only residues 589 to 666 assume coiled-coil conformation for dimer A/B; for dimer C/D, residues 578 to 666 are in a coiled-coil conformation. Note that both dimers have a peak in their inter-helical distance at the E613/R617/D620 junction. (D) Average B-factors for mainchain atoms at each residue for all four chains. Since the structure was refined using TLS, the B-factor plotted represents the isotropic equivalent of the total B-factor (B_tls + B_individual).

We analyzed the heptad register of ROCK-CC using the program SOCKET [32] based on the characteristic knobs-into-holes side-chain packing of a coiled-coil. ROCK-CC displays 11 consecutive heptad repeats starting at Leu-589 and ending at Lys-666. There are 12 layers of knobs-into-holes packing from chain A into B (and 13 layers from chain B into A) (Fig. 4A). At the coiled-coil interface, the *a* position knob from one molecule is inserted into a hole formed by four side chains at *d*-*g*-*a*-*d* positions of the neighboring molecule; and the *d* position knob is inserted into a hole formed by side chains at *a*-*d*-*e*-*a* positions of the neighbor.

The residues forming the heptads are plotted in a helical wheel showing the hydrophobic and polar interactions (Fig. 4B). In most coiled-coils, hydrophobic residues dominate at the *a* and *d* positions. In the ROCK-CC only about 50% of the *a* and *d* positions are taken by hydrophobic residues. Some of the *a* positions are occupied by arginine or lysine (Lys-596, Arg-652 and Lys-666), which use the long, aliphatic portions of their side chains to form hydrophobic contacts while the charged head groups are exposed to the solvent or make contacts with other residues on the surface. Previous studies have also shown that arginine or lysine can satisfy the requirement for hydrophobic residues in the heptad pattern [33], [34]. Hydrogen bonding also contributes to packing. Asn-589 at the *a* position and Ser-662 at the *d* position form hydrogen bonds across the coiled-coil interface to the same residues, with a bond distance of 2.7 and 2.8 Å respectively. Similar inter-coil interaction via hydrogen bond at the *d* position has also been observed in SNAP25 [35]. On the periphery of the structure, several distinct patterns of polar interactions are evident. There are two *a*-*g* interactions: Lys-596 of chain B forms a hydrogen bond to Ser-595 in chain A, and Lys-666 of chain B forms a salt bridge to Glu-665 of chain A (Fig. 4B). There are three *e*-*g* interactions: Gln-635 of chain A hydrogen bonds to Arg-630 of chain B, Lys-642 of chain A forms a salt bridge to Glu-637 of chain B, and Lys-642 of chain B forms a salt bridge to Glu-637 of chain A.

**Figure 5:**
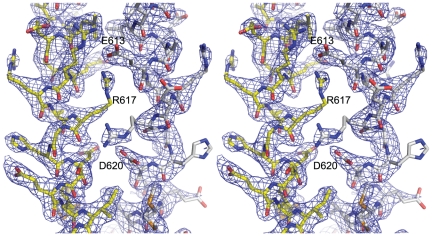
Stereogram of electron density map at the coiled-coil interface in the region of the E613/R617/D620 bulge. Chain C is shown in yellow and chain D in grey. The σ_A_ weighted 2F_O_ – F_C_ map is contoured at 1.5 σ and computed anisotropically to 2.3 Å. Due to the anisotropy, the map was sharpened by applying a B factor scaling of -10 Å^2^ (see methods).

Strikingly, near the center of the coiled-coil three consecutive *d*-*a*-*d* positions are occupied by charged residues. These residues (E613, R617 and D620) create an unusual bulge in the coiled-coil (Fig. 4B). Most interestingly, the charged head group of Arg-617 inserts into the center of the coiled-coil interface and together with Glu-613 and Asp-620, it appears to push apart the two helices (Fig. 5). The program TWISTER [36] was used to calculate the α-helical axes and the coiled-coil axis of ROCK-CC. The coiled-coil is bent by about 14° in the region centered around E613/R617/D620, where the inter-helical distance is also at its maximum (Fig. 4C). Inter-helical distance peaks at 11.2 Å on residue Glu-613. For comparison, the GCN4 leucine zipper (PDB code 2ZTA, 31 residues) [37] has an average inter-helical distance of 9.7 Å with a standard deviation of 0.26 Å.

We also examined this unique feature in dimer C/D, which is crystallographically distinct from dimer A/B. The electron density map (Fig. 5) at that region has slightly better quality than that of dimer A/B. In crystal lattice around R617, both chain B and D have fewer contacts to neighboring molecules than chain A or C. A thermal factor plot of all four chains showed chain B and D have much higher B factors than that of chain A and C in that region (Fig. 4D). Arg-617 of chain C forms one salt bridge each to Glu-613 of chain C and D. The positively charged arginine head group stabilizes these two negatively charged residues at the core *a*/*d* interface. The dimer interface is not symmetrical. Arg-617 of chain D forms an intra-helical salt bridge to Asp-620 and its carbonyl is distorted from an ideal α-helical geometry (Fig. 5). By comparison, Arg-617 of chain A forms the same interactions as in dimer C/D, but the side chain of Arg-617 of chain B is disordered (Fig. 4D). Thus the local conformation at the E613/R617/D620 junction is not precisely conserved between the dimers in the asymmetric unit. Additionally, the bend in their coiled-coil axes at the junction differs by ∼5°.

### Structural Similarity to Tropomyosin

**Figure 6:**
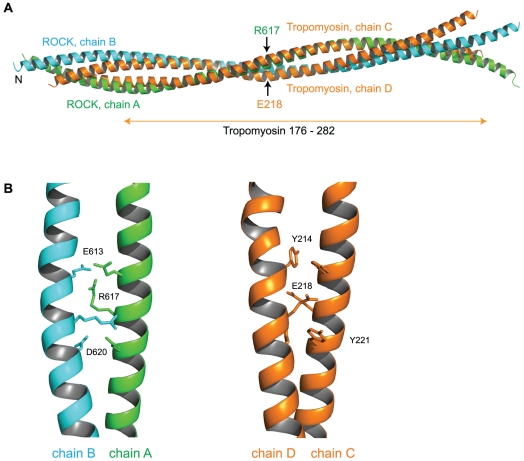
Superposition of ROCK-CC and tropomyosin (PDB code 2d3e). (A) DALI alignment matrix was based on only aligning ROCK-CC chain A to chain C in the tropomyosin structure. The tropomyosin structure is a fusion with the GCN4 leucine zipper; the tropomyosin portion of the structure (residues 176–282) is indicated below the superposition. Positions of R617 of ROCK chain A and E218 of tropomyosin chain D are indicated by arrows. (B) Detailed views of the respective unusual junctions in ROCK (left) and tropomyosin (right).

We used DALI [38] to search for proteins structurally related to ROCK-CC. The top match was rabbit skeletal muscle α tropomyosin (Fig. 6A). This tropomyosin structure was a fusion protein made by linking 23 residues of the leucine zipper from the yeast protein GCN4 with residues 176 to 282 of tropomyosin (PDB code 2d3e) [39]. The region of similarity includes the GCN4 fusion partner. Both structures are parallel two-stranded coiled-coils. Residues 552–681 of ROCK-CC superimpose with one of the chains of the “GCN4-tropomyosin” dimer with a rmsd deviation of 2.9 Å for 130 aligned C_α_ atoms and a Z score of 7.3. However, the sequence identity between tropomyosin residues 176–282 and corresponding ROCK is only 14%, so there is no clear evolutionary relationship between them. DALI also identified similarity with other coiled-coil proteins. Apart from tropomyosin structures, the next DALI match was Ndel1 (PDB code 2v71) [40], a coiled-coil protein that is required for organization of the cellular microtubule array and microtubule anchoring at the centrosome.

Structure of ROCK-CC is superimposed on tropomyosin in Fig. 6A. Interestingly tropomyosin has a discontinuity in its regular coiled-coil conformation at the same position as that found in ROCK-CC. This bulge is found by residues Y214/E218/Y221 in tropomyosin [39], [41] (Fig. 6). Like ROCK-CC, this tropomyosin bulge also causes a global bend by about 9° [39].

## Discussion

**Figure 7:**
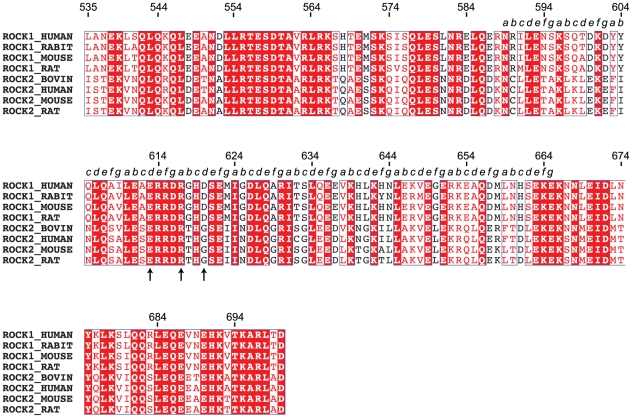
Sequence alignment of the ROCK coiled-coil region. Residues 535–700 is the region used for this crystallographic study. Identical residues are indicated by the red background, similar residues by red characters. The experimentally identified *a*-*g* heptad repeats are displayed above the sequence for residues 589 to 665. Residues E613, R617 and D620 are marked by arrows.

In this work, we describe the crystal structure of a tryptic fragment of the central coiled-coil domain of ROCK I. The structure reveals a bulge formed by residues E613, R617, D620 in the dimer. In a parallel coiled-coil dimer such as ROCK-CC, charged residues at the *a*/*d* core positions would be expected to create repulsion and instability. In ROCK-CC, these unusual residues are accommodated because the positively charged arginines (R617) are sandwiched between two layers of negatively charged residues (E613 and D620). Nevertheless, this unique sequence feature creates an irregularity in the coiled-coil structure. The conformation of these residues differs in the two dimers in the crystallographic asymmetric unit, which is suggestive of conformational flexibility or instability. This sequence irregularity is conserved; residues E613, R617, and D620 are identically conserved in ROCK I proteins of different species while D620 is replaced by glycine in ROCK II (Fig. 7). Gly-618 in ROCK I is also conserved. Glycine has high flexibility due to its lack of a side-chain and its placement close to R617 may facilitate backbone motion. In ROCK II, the conserved Gly-620 may provides the same type of flexibility. The conservation of this sequence irregularity suggests that it is likely to be functionally important.

ROCK proteins are thought to be autoinhibited by interactions between the N-terminal kinase domain and the C-terminal RBD and PH domains. This inhibitory interaction is expected to be released by binding of active Rho to the RBD [3], [5]. How does this autoinhibitory interaction occur if the relevant N- and C-terminal domains are separated by a rod-shaped coiled-coil domain over 220 Å in length? It is tempting to speculate that the E613/R617/D620 irregularity destabilizes the coiled-coil, effectively creating a hinge that allows the coiled-coil to “jackknife”. Simple geometric considerations reveal that a hinge at this central location could allow intramolecular autoinhibitory interactions to occur between the N-terminal kinase domain and C-terminal regulatory regions. Of course it is also possible that autoinhibition could occur in fully elongated ROCK molecules via formation of a head-to-tail dimer of dimers. Further biochemical characterization of autoinhibited ROCK will be required to distinguish between these models.

The functional significance, if any, of the structural similarity between ROCK-CC and tropomyosin is unclear. Tropomyosin polymerize head-to-tail along the actin filament. Because of the requirement to wind around the actin filament, tropomyosin has an undulating conformation and destabilized regions along its length to provide flexibility. There is high incidence of Ala at interface *d* positions, which are poorly packed and allow axial bends. Another mechanism of bending in tropomyosin is large, nonhydrophobic residues (such as Tyr or Glu) at the interface [39], [41]. Given the role of ROCK in regulating the actin cytoskeleton it will be of interest to determine whether it might bind directly to the actin filament via its coiled-coil region.

## Materials and Methods

### Protein Preparation

Various fragments encoding residues 535–741 of human p160 ROCK were PCR amplified from pBluescript-*myc*-p160 [11], and cloned into a modified pET30 vector that provides an N-terminal fused 6xHistidine-TEV tag. The plasmid was transformed into Rosetta2 (DE3) cells (Novagen) and grown at 37°C to an optical density of 0.5. The temperature was then shifted to 20°C and cells were induced with 0.25 mM IPTG for 15 h.

Initially, purified ROCK (535–741) protein was subjected to limited trypsin digest to define a construct suitable for crystallization. A fragment was produced whose limits were determined to be 535–709 by HPLC-ESI-MS (Thermo Fisher Scientific LTQ Orbitrap XL mass spectrometer). ROCK (535–709), expressed as described above, readily yielded crystals in space group *P*1 that proved resistant to structure determination (see below). However, a slightly shorter construct (535–700) yielded crystals in a tetragonal space group that proved amenable to structure determination.

Cells bearing ROCK (535–700) were lysed by sonication in lysis buffer (20 mM Tris, pH 8.0/300 mM KCl/10% glycerol/40 mM imidazole/10 mM 2-mercaptoethanol/1 mM PMSF) and cleared by high-speed centrifugation. The supernatant was incubated with Ni-NTA agarose (Qiagen) for 3 h at 4°C, washed, and the protein was eluted by elution buffer (20 mM Tris, pH 8.0/500 mM imidazole/300 mM KCl/10% glycerol/10 mM 2-mercaptoethanol). TEV protease was added to remove the 6xHistidine tag at 4°C overnight. Proteins were diluted 1∶10 into cation-exchange buffer A (50 mM MES, pH 6.0/1 mM DTT) and loaded onto 2×5 ml HiTrap SP HP columns (GE Healthcare). A gradient of 0–100% buffer B (50 mM MES, pH 6.0/1M KCl/1 mM DTT) over 20 column volume was applied and ROCK eluted from abount 75 to 225 mM KCl in a broad peak. The purified protein was dialyzed against final buffer (20 mM Hepes, pH 7.5/100 mM NaCl/5 mM DTT), concentrated to 10 mg/ml, and stored at −80°C. Selenomethionine (SeMet) protein was obtained as above, except for the following: SeMet was incorporated by metabolic inhibition [42], 20 mM 2-mercaptoethanol was added in the lysis buffer, and after Ni-NTA elution step all buffers contained 10 mM DTT.

### Crystallization, Data collection, and Processing

Initial screens of crystallization conditions were carried out by using 96-well format Index and Crystal Screen HT (Hampton Research), and Classics Lite (Qiagen) on a Phoenix robot (Art Robbins Instruments). Index kit condition #56 [0.2 M KCl/50 mM Hepes, pH 7.5/35% (v/v) Pentaerythritol propoxylate (5/4 PO/OH)] produced initial hit for ROCK (535–709). After optimization, crystals of ROCK (535–709) were grown by hanging-drop vapor diffusion at 20°C. 2 µl of 10 mg/ml protein was mixed with an equal volume of mother liquid consisting of 29% (v/v) Pentaerythritol propoxylate (5/4 PO/OH), 240 mM KCl, 100 mM MES (pH 6.8), and 5 mM DTT and equilibrated against 0.5 ml of mother liquor. Crystals grew as thin plates to full size (≈0.2 mm ×0.1 mm ×0.02 mm) in 3 weeks and were directly frozen out of mother liquor into liquid nitrogen. A native data set to 2.40 Å (Table 1) was collected at CHESS F2 beamline at Cornel High Energy Synchrotron Source. However, various phasing attempts including SAD/MAD on SeMet labeled crystals and SIRAS/SAD/MAD on K_2_PtCl_4_ soaks all failed. Crystals of ROCK (535–709) formed in space group *P*1 and displayed strong non-origin peaks in its Native Patterson map. The spacing between the peaks (5.2 Å) indicated that these peaks arose from intra-helical vectors of long coiled-coil structures all aligned in one direction. Therefore efforts were directed in finding an alternate space group.

Classics Lite kit condition #16 [0.2 M NaCl/100 mM Na acetate, pH 4.6/15% (v/v) MPD] gave initial hit for ROCK (535–700) and the crystal was found to be in space group *P*4_3_2_1_2. SeMet ROCK (535–700) protein was crystallized in condition consisting of 13% MPD, 100 mM NaCl, 100 mM sodium acetate (pH 4.9), 10 mM DTT, 5% glycerol and 10 mM spermidine. Crystals grew as tetragonal rods to full size (≈0.5 mm ×0.1 mm ×0.1 mm) in 10 days and were directly frozen out of mother liquor into liquid nitrogen. A highly redundant SAD data set was collected at the peak wavelength at the NE-CAT 24ID-C beamline at Argonne National Laboratory. Integration, scaling, and merging of the diffraction data were performed with HKL2000 [43]. The diffraction pattern generated by ROCK (535–700) SeMet crystals exhibited anisotropy, which had strong diffraction to *d*_min_ = 2.3 Å along c*, but only weakly to 2.9 Å along a* and b*. Therefore a custom FORTRAN program was used to filter the data in an ellipsoid along *h*_max_ = 29, *k*_max_ = 29, and *l*_max_ = 162 by the formula (*h* ^2^/*h*_max_^2^ + *k* ^2^/*k*_max_^2^ + *l* ^2^/*l*_max_^2^) ≤1. Data filtering was done on HKL2000. X files, which were integrated in a 2.3 Å sphere. A summary of the data collection statistics is given in Table 1 and 2. For comparison, data collection statistics without ellipsoidal filtering is given in Table 3.

### Structure Determination

The substructure determination, phasing, density modification and initial automatic model building were all performed by using the AutoSol Wizard of Phenix[44]. Twelve selenium were initially identified (arising from 12 selenomethionine residues) and the Phaser SAD phasing module [45] of Phenix AutoSol extended it to 36 sites (the additional sites arise from alternate SeMet side chain conformations). Because of the anisotropy, electron density maps calculated by Phaser were automatically sharpened. Density modification and automatic tracing in the Resolve module [46], [47] of Phenix AutoSol produced a starting model that assigned 52% of the side chains and 84% of total residues. The structure was completed by iterative model building in Coot [48] and refinement in CNS [49] consisting of torsion angle molecular dynamics simulated annealing, and individual restrained thermal factor refinement. A final round of refinement was performed using Phenix incorporating TLS parameters that defined each ROCK chain as a pseudo-rigid body. The final model consists of four chains A, B, C and D with residues 542–693, 541–692, 535–692, and 535–693 respectively, and 27 water molecules. NCS restraints were not used during refinement. Model statistics are shown in Table 1. The atomic coordinates and diffraction data have been deposited in the Protein Data Bank [ID code 3O0Z].

### Structure Analysis

Using the program TWISTER [36], the center of each α-helix, O_n_ at residue n was calculated by five surrounding C_α_ atoms from n - 2 to n + 2. The coiled-coil axis is defined as the geometric average of the O_n_ positions. Helical distance (HD) is the distance between two α-helical centers at residue n. The program SOCKET [29] was used to define the beginning and end of coiled-coil motif.

### Multi-Angle Light Scattering Analysis

Purified ROCK (535–704) was separated on a Superdex 200 column (GE Healthcare) pre-equilibrated with 20 mM Hepes pH 7.5, 150 mM NaCl, 2 mM TCEP, and connected to a Wyatt miniDAWN TREOS three-angle light scattering detector and Optilab-rEX refractive index detector.

## References

[1] Nakagawa O, Fujisawa K, Ishizaki T, Saito Y, Nakao K (1996). ROCK-I and ROCK-II, two isoforms of Rho-associated coiled-coil forming protein serine/threonine kinase in mice.. FEBS Lett.

[2] Leung T, Chen XQ, Manser E, Lim L (1996). The p160 RhoA-binding kinase ROK alpha is a member of a kinase family and is involved in the reorganization of the cytoskeleton.. Mol Cell Biol.

[3] Ishizaki T, Maekawa M, Fujisawa K, Okawa K, Iwamatsu A (1996). The small GTP-binding protein Rho binds to and activates a 160 kDa Ser/Thr protein kinase homologous to myotonic dystrophy kinase.. EMBO J.

[4] Leung T, Manser E, Tan L, Lim L (1995). A novel serine/threonine kinase binding the Ras-related RhoA GTPase which translocates the kinase to peripheral membranes.. J Biol Chem.

[5] Matsui T, Amano M, Yamamoto T, Chihara K, Nakafuku M (1996). Rho-associated kinase, a novel serine/threonine kinase, as a putative target for small GTP binding protein Rho.. EMBO J.

[6] Riento K, Ridley AJ (2003). Rocks: multifunctional kinases in cell behaviour.. Nat Rev Mol Cell Biol.

[7] Amano M, Ito M, Kimura K, Fukata Y, Chihara K (1996). Phosphorylation and activation of myosin by Rho-associated kinase (Rho-kinase).. J Biol Chem.

[8] Kawano Y, Fukata Y, Oshiro N, Amano M, Nakamura T (1999). Phosphorylation of myosin-binding subunit (MBS) of myosin phosphatase by Rho-kinase in vivo.. J Cell Biol.

[9] Kureishi Y, Kobayashi S, Amano M, Kimura K, Kanaide H (1997). Rho-associated kinase directly induces smooth muscle contraction through myosin light chain phosphorylation.. J Biol Chem.

[10] Amano M, Chihara K, Kimura K, Fukata Y, Nakamura N (1997). Formation of actin stress fibers and focal adhesions enhanced by Rho-kinase.. Science.

[11] Ishizaki T, Naito M, Fujisawa K, Maekawa M, Watanabe N (1997). p160ROCK, a Rho-associated coiled-coil forming protein kinase, works downstream of Rho and induces focal adhesions.. FEBS Lett.

[12] Amano M, Chihara K, Nakamura N, Fukata Y, Yano T (1998). Myosin II activation promotes neurite retraction during the action of Rho and Rho-kinase.. Genes Cells.

[13] Fukata Y, Oshiro N, Kinoshita N, Kawano Y, Matsuoka Y (1999). Phosphorylation of adducin by Rho-kinase plays a crucial role in cell motility.. J Cell Biol.

[14] Chrzanowska-Wodnicka M, Burridge K (1996). Rho-stimulated contractility drives the formation of stress fibers and focal adhesions.. J Cell Biol.

[15] Maekawa M, Ishizaki T, Boku S, Watanabe N, Fujita A (1999). Signaling from Rho to the actin cytoskeleton through protein kinases ROCK and LIM-kinase.. Science.

[16] Li F, Higgs HN (2003). The mouse Formin mDia1 is a potent actin nucleation factor regulated by autoinhibition.. Curr Biol.

[17] Watanabe N, Kato T, Fujita A, Ishizaki T, Narumiya S (1999). Cooperation between mDia1 and ROCK in Rho-induced actin reorganization.. Nature Cell Biology.

[18] Watanabe S, Ando Y, Yasuda S, Hosoya H, Watanabe N (2008). mDia2 induces the actin scaffold for the contractile ring and stabilizes its position during cytokinesis in NIH 3T3 cells.. Mol Biol Cell.

[19] Kosako H, Yoshida T, Matsumura F, Ishizaki T, Narumiya S (2000). Rho-kinase/ROCK is involved in cytokinesis through the phosphorylation of myosin light chain and not ezrin/radixin/moesin proteins at the cleavage furrow.. Oncogene.

[20] Mukai Y, Shimokawa H, Matoba T, Kandabashi T, Satoh S (2001). Involvement of Rho-kinase in hypertensive vascular disease: a novel therapeutic target in hypertension.. FASEB J.

[21] Sato M, Tani E, Fujikawa H, Kaibuchi K (2000). Involvement of Rho-kinase-mediated phosphorylation of myosin light chain in enhancement of cerebral vasospasm.. Circ Res.

[22] Chiba Y, Takada Y, Miyamoto S, MitsuiSaito M, Karaki H (1999). Augmented acetylcholine-induced, Rho-mediated Ca2+ sensitization of bronchial smooth muscle contraction in antigen-induced airway hyperresponsive rats.. Br J Pharmacol.

[23] Itoh K, Yoshioka K, Akedo H, Uehata M, Ishizaki T (1999). An essential part for Rho-associated kinase in the transcellular invasion of tumor cells.. Nat Med.

[24] Nakajima M, Hayashi K, Katayama K, Amano Y, Egi Y (2003). Wf-536 prevents tumor metastasis by inhibiting both tumor motility and angiogenic actions.. Eur J Pharmacol.

[25] Dvorsky R, Blumenstein L, Vetter IR, Ahmadian MR (2004). Structural insights into the interaction of ROCKI with the switch regions of RhoA.. J Biol Chem.

[26] Amano M, Chihara K, Nakamura N, Kaneko T, Matsuura Y (1999). The COOH terminus of Rho-kinase negatively regulates rho-kinase activity.. J Biol Chem.

[27] Sebbagh M, Renvoize C, Hamelin J, Riche N, Bertoglio J (2001). Caspase-3-mediated cleavage of ROCK I induces MLC phosphorylation and apoptotic membrane blebbing.. Nat Cell Biol.

[28] Coleman ML, Sahai EA, Yeo M, Bosch M, Dewar A (2001). Membrane blebbing during apoptosis results from caspase-mediated activation of ROCK I.. Nat Cell Biol.

[29] Jacobs M, Hayakawa K, Swenson L, Bellon S, Fleming M (2006). The structure of dimeric ROCK I reveals the mechanism for ligand selectivity.. Journal of Biological Chemistry.

[30] Lupas A, Van Dyke M, Stock J (1991). Predicting coiled coils from protein sequences.. Science.

[31] Burkhard P, Stetefeld J, Strelkov SV (2001). Coiled coils: a highly versatile protein folding motif.. Trends Cell Biol.

[32] Walshaw J, Woolfson DN (2001). Socket: a program for identifying and analysing coiled-coil motifs within protein structures.. J Mol Biol.

[33] Grum VL, Li D, MacDonald RI, Mondragon A (1999). Structures of two repeats of spectrin suggest models of flexibility.. Cell.

[34] Glover JN, Harrison SC (1995). Crystal structure of the heterodimeric bZIP transcription factor c-Fos-c-Jun bound to DNA.. Nature.

[35] Freedman SJ, Song HK, Xu Y, Sun ZY, Eck MJ (2003). Homotetrameric structure of the SNAP-23 N-terminal coiled-coil domain.. J Biol Chem.

[36] Strelkov SV, Burkhard P (2002). Analysis of alpha-helical coiled coils with the program TWISTER reveals a structural mechanism for stutter compensation.. J Struct Biol.

[37] O'Shea EK, Klemm JD, Kim PS, Alber T (1991). X-ray structure of the GCN4 leucine zipper, a two-stranded, parallel coiled coil.. Science.

[38] Holm L, Sander C (1993). Protein structure comparison by alignment of distance matrices.. J Mol Biol.

[39] Nitanai Y, Minakata S, Maeda K, Oda N, Maeda Y (2007). Crystal structures of tropomyosin: flexible coiled-coil.. Adv Exp Med Biol.

[40] Derewenda U, Tarricone C, Choi WC, Cooper DR, Lukasik S (2007). The structure of the coiled-coil domain of Ndel1 and the basis of its interaction with Lis1, the causal protein of Miller-Dieker lissencephaly.. Structure.

[41] Whitby FG, Phillips GN (2000). Crystal structure of tropomyosin at 7 Angstroms resolution.. Proteins.

[42] Van Duyne GD, Standaert RF, Karplus PA, Schreiber SL, Clardy J (1993). Atomic structures of the human immunophilin FKBP-12 complexes with FK506 and rapamycin.. Journal of molecular biology.

[43] Otwinowski Z, Minor W (1997). Processing of X-ray diffraction data collected in oscillation mode.. Methods in enzymology.

[44] Adams PD, Afonine PV, Bunkoczi G, Chen VB, Davis IW (2010). PHENIX: a comprehensive Python-based system for macromolecular structure solution.. Acta Crystallogr D Biol Crystallogr.

[45] McCoy AJ, Grosse-Kunstleve RW, Adams PD, Winn MD, Storoni LC (2007). Phaser crystallographic software.. J Appl Crystallogr.

[46] Terwilliger TC (2003). Automated main-chain model building by template matching and iterative fragment extension.. Acta Crystallogr D Biol Crystallogr.

[47] Terwilliger TC (2000). Maximum-likelihood density modification.. Acta Crystallogr D Biol Crystallogr.

[48] Emsley P, Cowtan K (2004). Coot: model-building tools for molecular graphics.. Acta Crystallographica Section D: Biological Crystallography.

[49] Brunger AT (2007). Version 1.2 of the Crystallography and NMR system.. Nature Protocols.

